# A Bayesian framework to integrate multi-level genome-scale data for Autism risk gene prioritization

**DOI:** 10.1186/s12859-022-04616-y

**Published:** 2022-04-22

**Authors:** Ying Ji, Rui Chen, Quan Wang, Qiang Wei, Ran Tao, Bingshan Li

**Affiliations:** 1 Department of Molecular Physiology and Biophysics, Vanderbilt University, Nashville, TN 37212 USA; 2 Vanderbilt Genetics Institute, Vanderbilt University Medical Center, Nashville, TN 37212 USA; 3 Department of Biostatistics, Vanderbilt University, Nashville, TN 37212 USA

**Keywords:** Gene prioritization, Bayesian model selection, ASD risk genes

## Abstract

**Background:**

Autism spectrum disorder (ASD) is a group of complex neurodevelopment disorders with a strong genetic basis. Large scale sequencing studies have identified over one hundred ASD risk genes. Nevertheless, the vast majority of ASD risk genes remain to be discovered, as it is estimated that more than 1000 genes are likely to be involved in ASD risk. Prioritization of risk genes is an effective strategy to increase the power of identifying novel risk genes in genetics studies of ASD. As ASD risk genes are likely to exhibit distinct properties from multiple angles, we reason that integrating multiple levels of genomic data is a powerful approach to pinpoint genuine ASD risk genes.

**Results:**

We present BNScore, a Bayesian model selection framework to probabilistically prioritize ASD risk genes through explicitly integrating evidence from sequencing-identified ASD genes, biological annotations, and gene functional network. We demonstrate the validity of our approach and its improved performance over existing methods by examining the resulting top candidate ASD risk genes against sets of high-confidence benchmark genes and large-scale ASD genome-wide association studies. We assess the tissue-, cell type- and development stage-specific expression properties of top prioritized genes, and find strong expression specificity in brain tissues, striatal medium spiny neurons, and fetal developmental stages.

**Conclusions:**

In summary, we show that by integrating sequencing findings, functional annotation profiles, and gene-gene functional network, our proposed BNScore provides competitive performance compared to current state-of-the-art methods in prioritizing ASD genes. Our method offers a general and flexible strategy to risk gene prioritization that can potentially be applied to other complex traits as well.

**Supplementary Information:**

The online version contains supplementary material available at 10.1186/s12859-022-04616-y.

## Background

Genetics plays an important role in the etiology of Autism spectrum disorder (ASD). Dozens of ASD risk genes have been identified from whole exome sequencing (WES) studies (e.g., de novo and inherited mutations) [1–3], but the vast majority of ASD risk genes remains unknown, as it has been estimated that more than 1000 genes are involved in risk of ASD [4]. It is challenging to identify ASD risk genes due to its broad spectrum of genetic architectures with multiple biological processes involved [5]. WES or whole genome-sequencing (WGS) studies of parent-offspring trios has been successful in identifying ASD risk genes via de novo mutations. However, the power of such approaches is inevitably low given the rarity of de novo mutations. Computational approaches provide cost-effective alternatives to effectively nominate ASD risk genes [6].

Various computational methods have been developed for ASD risk gene identification, most of which employ diverse biological evidence to prioritize risk genes [4, 7, 8]. Conceptually, a candidate gene is often scored higher if the gene is similar or closer to known ASD risk genes (referred to as “seed genes” hereafter) based on the biological evidence. In practice, those approaches start by bringing together a “gold-standard” seed gene set, then train a statistical model using seed genes along with their relevant biological evidence, and finally rank all genes based on the predicted scores from the trained model. Two key ingredients to facilitate successful modeling are (1) a robust seed gene set comprising true ASD risk genes and (2) relevant biological evidence that are representative of these genes.

The biological evidence for prioritization mainly pertains to genetic sequence properties, functional annotation, and network information [9, 10]. A few recent studies are based on one or two types of the aforementioned evidence. He et al. [4] prioritized genes through genetic sequence properties alone (e.g., multiple occurrences of mutations in unrelated patients) and didn’t consider other evidence that are important to ASD risk. Functional annotation based methods [11, 12] rank genes according to the similarity between candidate and seed genes’ annotation profiles. They tend to bias towards well-annotated genes and are less effective for genome-wide prediction [13]. Network-based approaches [7, 8, 14, 15] rely on network proximity between candidate and seed genes, thus are less biased to well-annotated genes. Instead, the results may bias towards highly connected genes. Recently, an increasing proportion of network-based approaches are framing the prioritization problem as a classification problem (i.e., classify genes into ASD risk genes versus non-risk genes) to be solved by machine-learning algorithms [7, 8]. Apart from traditional machine learning approaches, deep learning has also been rapidly gaining popularity. Graph neural network (GNN) can directly analyze data structured as graphs, such as biological networks. Zhang et al. [15] recent applied a GNN classifier to prioritize ASD genes using the human molecular interaction network input for training and reported to outperform other commonly used machine learning algorithms. Apart from efforts based on network only, Lin et al. [16] integrated network evidence with other biological evidence using machine learning classifiers for risk gene prioritization. These machine-learning based methods have discovered novel ASD genes, but their inherent “black box” nature limits the interpretability of the final results.

Herein, we present BNScore, a novel Bayesian model selection approach for ASD risk gene prioritization through explicitly integrating three major types of biological evidence: (1) seed genes derived from ASD sequencing studies; (2) multiple lines of gene-level functional annotations; and (3) distance to known ASD risk genes in a biological network. The framework is flexible in that it can readily include additional features relevant to ASD to further increase prediction accuracy. In addition, the Bayesian set-up renders a clear interpretation of the prediction scores (i.e., Bayesian posterior odds of being a risk gene versus not) for each gene across the genome. We demonstrate the validity of our approach and its improved performance over existing methods by examining the resulting top candidate ASD risk genes against sets of high-confidence benchmark genes and large scale ASD genome-wide association studies (GWAS). We study the brain spatiotemporal gene expression specificity of identified top candidate genes to implicate tissues, cell types, and development stages in the etiology of ASD.

## Methods

We frame the task of finding risk genes as a Bayesian model selection problem. For each gene, we select between models $M_{0}$, not a ASD risk gene and $M_{1}$, a risk gene. Let $\varvec{\theta _{j}}$ denote the parameters associated with model $M_{j}$ ($j=1,0$) and let $\varvec{D} = (D_{1},D_{2},...,D_{p})^\mathrm{T}$ denote the *p*-dimensional functional annotation data for each gene. We formulate the posterior odds of a gene being a risk gene as

$$\begin{aligned} \frac{P(M_{1}|\varvec{D})}{P(M_{0}|\varvec{D})}&= \frac{P(M_{1})P(\varvec{D}|M_{1})}{P(M_{0})P(\varvec{D}|M_{0})} \\&= \frac{P(M_{1})}{P(M_{0})}\frac{\int p(\varvec{D}|\varvec{\theta _{1}}, M_{1})p(\varvec{\theta _{1}}|M_{1})d\varvec{\theta _{1}}}{\int p(\varvec{D}|\varvec{\theta _{0}}, M_{0})p(\varvec{\theta _{0}}|M_{0})d\varvec{\theta _{0}}}, \end{aligned}$$

where $\frac{P(M_{1})}{P(M_{0})}$ and $\frac{P(\varvec{D}|M_{1})}{P(\varvec{D}|M_{0})}$ are the prior odds and Bayes factor of being a risk gene, respectively. For each gene, we specify $\frac{P(M_{1})}{P(M_{0})}$ based on its average distance to seed genes in a gene-gene functional network denoted by $N_{S}$, based on the rationale that disease-associated genes are assumed to be closer to each other than random pairs in the network [5, 8, 11]. We assume that the annotations in $\varvec{D}$ are independent of each other under both $M_{0}$ and $M_{1}$ and compute gene-level Bayes factor $\frac{P(\varvec{D}|M_{1})}{P(\varvec{D}|M_{0})}$ by taking the product of the Bayes factors from individual annotations, i.e., $\frac{P(\varvec{D}|M_{1})}{P(\varvec{D}|M_{0})}=\prod _{l=1}^{p}\frac{P(D_{l}|M_{1})}{P(D_{l}|M_{0})}$.

**Fig. 1:**
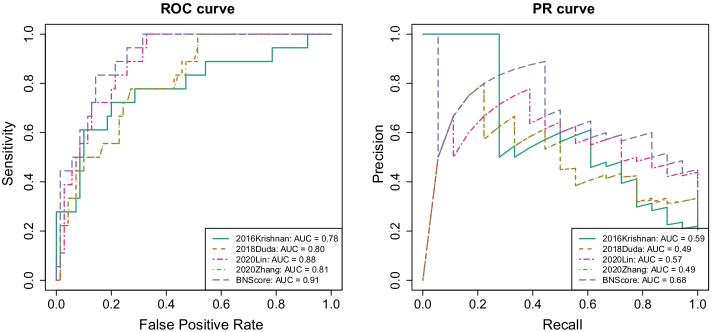
ROC and PR curves of the four methods in the ASD2020 gene set

**Fig. 2:**
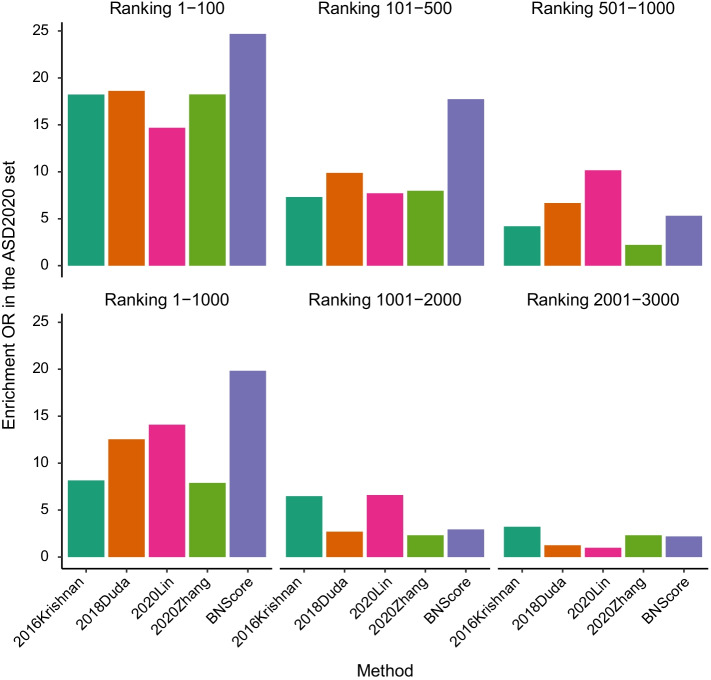
Enrichment of top candidate genes in the ASD2020 gene set

**Fig. 3:**
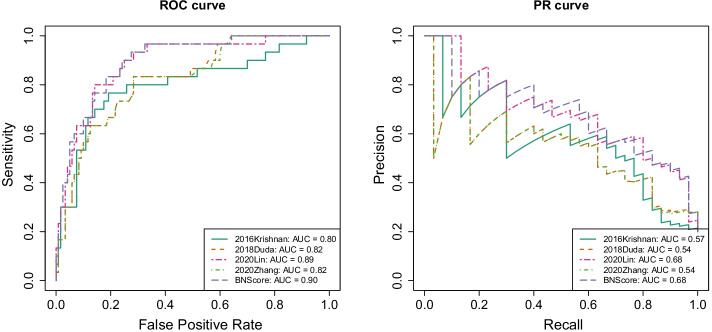
ROC and PR curves of the four methods in the SFARI T1 gene set

**Fig. 4:**
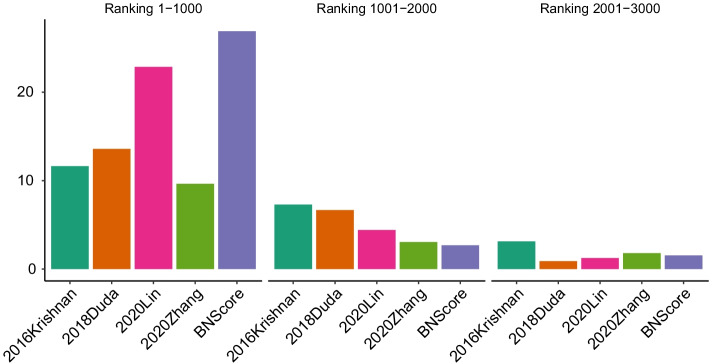
Enrichment of top candidate genes in the SFARI T1 gene set

**Fig. 5:**
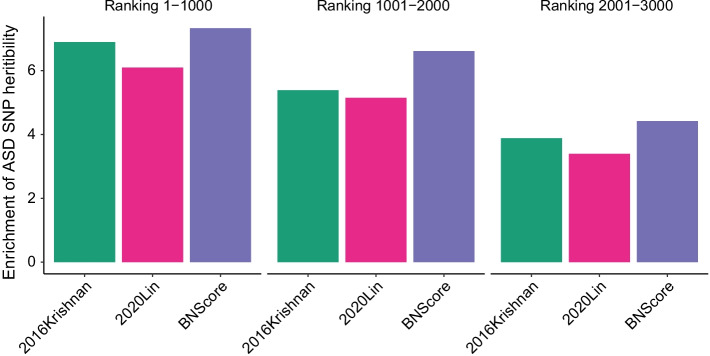
Enrichment of ASD heritability for top candidate genes

In our framework, seed genes and “background” genes are essential to derive Bayes factors and prior odds. We use 65 genes identified from a large exome sequencing study as seed genes [3]. We randomly select 500 genes across the genome (excluding the 65 seed genes) and regard them as background genes. We acknowledge that these background genes are not strictly non-ASD genes. However, there is no gold standard for non-ASD risk genes and it is reasonable to assume that the vast majority of genes across the genome are true non-ASD genes. There may be a few true positive genes ended up in our background gene set, in which situation the resulting inference could be slightly conservative. Nevertheless, we believe that this strategy is more robust than using an unreliable “gold-standard” background gene set. We choose 500 background genes by trial and error to balance between two considerations: a smaller set may be insufficiently representative of the genome background and a larger set can be too heterogeneous to be useful [17].

### Prior odds of a gene being an ASD risk gene

We assume that the prior probability of a gene being a risk gene is determined by two factors: (1) the overall fraction of risk genes in the genome and (2) the average distance of this gene to seed genes in a gene-gene functional network. The rationale is that the closer the two genes are in the network, the higher chance that they have similar functions. We generate the prior odds from

$$\begin{aligned} \frac{P(M_{1})}{P(M_{0})} = \frac{1000}{18000-1000}P(N_{s}), \end{aligned}$$

where $\frac{1000}{18{,}000{-}1000}$ is the assumed constant ratio of risk versus non-risk genes in the genome, as it’s estimated that around 1000 genes in 18000 genes are ASD risk genes [4]. $P(N_{s})$ is the average distance between the current gene and all seed genes, which is calculated based on Gene Ontology (GO) [18, 19]. Specifically, we first build a network connecting all pairs of genes based on the number and strength of GO terms shared by each gene pair. In this network, the distance between any gene pair is proportional to the log likelihood ratio of the two genes sharing the same GO annotations versus not [14].

We then construct a transition matrix from the network and apply the random walk with restart algorithm [20] to derive the reaching probabilities between any pair of genes.

Finally, we calculate $P(N_{s})$ as the average reaching probabilities of the current gene to all seed genes (see Additional file 1: Section A of Supplementary Notes for details).

### Bayes factor of a gene being an ASD risk gene

To reflect each gene’s strength of ASD association from a collection of functional annotations, we first identify ASD related biological processes and then summarize the ensemble evidence in a Bayes factor. We consider two forms of functional annotations: (1) binary annotation: presence/absence in biological processes previously implicated in ASD (e.g., genes involved in the developmental processes, which have been reported to be important in the pathogenesis of ASD [21]); and (2) continuous annotation: gene-level metrics (e.g., probability of being loss-of-function (LoF) intolerant (pLI) score [22]). We model binary and continuous annotations using Beta-Bernoulli and Normal-Inverse Gamma distributions, respectively [23]. We specify the prior distributions $p(\varvec{\theta _{1}}|M_{1})$ and $p(\varvec{\theta _{0}}|M_{0})$ via parametric Empirical Bayes approaches using seed and background genes (see Additional file 1: Section B of Supplementary Notes for details).

#### ASD related functional annotations

We initially collect 61 ASD related binary and continuous annotations from literature and then remove redundant or irrelevant annotations using seed and background genes. Specifically, we use Fisher’s exact test to identify binary annotations enriched for seed genes, and we use t-test to identify continuous annotations with significant differences between seed and background genes. The selected annotations consist of (1) biological processes implicated in ASD, e.g., genes encoding chromatin modifiers, (2) important regulatory targets, e.g., targets of FMRP, which is a polyribosome-associated RNA binding protein that plays important roles in synaptic function and neuronal plasticity, and (3) generic gene-level metrics, e.g., the pLI score; see Additional file 1: Table S1 for a complete list of annotations.

#### Computing Bayes factor based on a binary annotation

We assume that a binary annotation $D_l$ follows a Bernoulli distribution $Bernoulli(\theta _{lj})$, where $\theta _{lj}$ represents the fraction of disease-associated genes with this annotation under model $M_j$ $(j=0,1)$. We assume the prior distribution of $\theta _{lj}$ to be $Beta(\alpha _{lj},\beta _{lj})$, where $\alpha _{lj}$ and $\beta _{lj}$ are hyperparameters. Suppose we have *n* seed genes, *k* of which possess this binary annotation, i.e., $\sum _{g=1}^{n} D_{lg} = k$. It can be shown that the marginal distribution of $D_{l}$ under model $M_j$ is $p(D_{l}|\alpha _{lj},\beta _{lj}) = \int p(D_{l}|\theta _{lj})p(\theta _{lj}|\alpha _{lj},\beta _{lj})d\theta _{lj} = \frac{B(\alpha _{lj} + k, n-k+\beta _{lj})}{ B(\alpha _{lj},\beta _{lj})}$, where $B(\cdot ,\cdot )$ is the beta function. We obtain moment estimators of hyperparameters $\widetilde{\alpha }_{lj}$ and $\widetilde{\beta }_{lj}$ under $M_{j}$ using the seed and background genes (see Additional file 1: Section B.1 of Supplementary Notes for details). Then, for each candidate gene (i.e., any gene not in the seed and background gene sets), we determine which model gives a better fit using the Bayes factor $\frac{p(D_{l}|M_{1})}{p(D_{l}|M_{0})} = \frac{p(D_{l}|\widetilde{\alpha }_{l1}, \widetilde{\beta }_{l1})}{p(D_{l}|\widetilde{\alpha }_{l0}, \widetilde{\beta }_{l0})}$.

#### Computing Bayes factor based on a continuous annotation

We assume that a continuous annotation $D_{l}$ follows a normal distribution $N(\mu _{lj}, \theta _{lj})$, where $\mu _{lj}$ and $\theta _{lj}$ are the mean and variance, respectively, under model $M_j$ $(j = 0, 1)$. We assume a Normal-Inverse Gamma prior for $\mu _{lj}$ and $\theta _{lj}$, i.e., $\mu _{lj}|\theta _{lj} \sim N(\mu _{0lj},\frac{\theta _{lj}}{\kappa _{lj}})$; $\theta _{lj} \sim IG(\upsilon _{lj}/2,\upsilon _{lj}\sigma _{lj}^{2}/2)$. Let $\varvec{\eta _{lj}}=(\mu _{0lj},\kappa _{lj},\upsilon _{lj},\sigma _{lj}^{2})^\mathrm{T}$ denote the hyperparameters. The marginal distribution of $D_{l}$ under model $M_j$ is $p(D_{l}|\varvec{\eta _{lj}})=\int p(D_{l}|\mu _{lj},\theta _{lj}) p(\mu _{lj}|\mu _{0lj},\theta _{lj},\kappa _{lj})p(\theta _{lj}|\upsilon _{lj},\sigma ^2_{lj})d\mu _{lj}d\theta _{lj}$, which can be shown to be a non-standardized t distribution $t_{\upsilon _{lj}}(\mu _{0lj},\sigma _{lj}^{2}(1+\kappa _{lj})/\kappa _{lj})$ [24]. We obtain moment estimators of hyperparameters $\widetilde{\varvec{\eta }}_{lj}$ under $M_{j}$ using the seed and background genes (see Additional file 1: Section B.2 of Supplementary Notes for details). Then, for each candidate gene, we determine which model gives a better fit using the Bayes factor $\frac{p(D_{l}|M_{1})}{p(D_{l}|M_{0})} = \frac{p(D_{l}|\widetilde{\varvec{\eta }}_{l1})}{p(D_{l}|\widetilde{\varvec{\eta }}_{l0})}$.

### Comparing with existing methods for ASD gene prioritization

We compare our method with three state-of-the-art methods developed by Krishnan et al. [8] (referred to as “2016Krishnan”), Duda et al. [7] (referred to as “2018Duda”), Lin et al. [16] (referred to as “2020Lin”), and Zhang et al. [15] (referred to as “2020Zhang”). These methods used machine learning algorithms with different types of evidence: 2016Krishnan utilized a brain-specific functional network reflecting brain-specific expression and biological processes; 2018Duda utilized publicly available tissue-specific microarray, protein interaction, and phenotype annotation data sets; 2020Lin utilized spatiotemporal gene expression patterns in human brain, gene-level constraint metrics, and other gene variation features; 2020Zhang utilized a human molecular interaction network based on literature of physical protein interactions experimentally documented. These methods used different seed and background gene sets for training but they all include the 65 seed genes described previously. We use their gene prioritization scores directly rather than retraining the models. For fair comparison, we depleted the training genes and only evaluate the testing genes for each method using the external benchmarks. Specifically, we obtain the 2016Krishnan, 2018Duda, 2020Lin, and 2020Zhang scores from Supplementary Table 3 of Krishnan et al. [8], Supplemental Table 1 of Duda et al. [7], and Supplemental Table 3 of Lin et al. [16], Github repository [25] by Zhang et al. [15].

### Measure of gene expression specificity

We use the specificity index (*SI* and *pSI*) defined by Dougherty et al. [26] to measure expression specificity of candidate genes under various biological conditions (i.e., tissue, cell type, brain region, and developmental stage). Suppose there are *m* potential conditions. We want to calculate the *SI* for gene *g* under the first condition compared to the other $m-1$ conditions. Let $E_{1,g}$ denote the expression of gene *g* under the first condition. We compute a fold-change value for gene *g* to measure its relative expression under the first condition to the $k\hbox {th}$ condition and obtain the rank of this fold change value $R_{1/k,g}$ relative to other genes. Then, we define *SI* for gene *g* as the average rank of $R_{1/k,g}$ under all conditions, i.e., $SI_{g,1} = \sum _{k=2}^{m}R_{1/k,g}/(m-1)$. Raw *SI* scores are not directly comparable across conditions due to the differences in number of genes expressed under each condition, so *pSI*, a permutation based *p* value for each *SI* is computed by randomly shuffling expression values and computing *SI* to determine the probability of observing a *SI* value less than or equal to a predefined threshold of 0.05 in the permutated distribution. The *pSI* value is used to assess gene expression specification under certain conditions. The code used to calculate *SI* and *pSI* was obtained from Dougherty lab website [27].

## Results

### Benchmarking using sequencing-identified novel ASD genes

We obtained 102 ASD genes identified in a recently published large exome sequencing study [28] (referred to as the “2020 study”). Among these 102 ASD genes, 65 had been previously identified in Sanders et al. [3] (referred to as the “2015 study”). These two studies enable us to conduct a “time-lapse” data experiment [29]. That is, we prioritized ASD risk genes based on the 65 seed genes identified in the 2015 study, and then evaluated the top candidate genes against those identified in the 2020 study but not in the 2015 study (referred to as the “ASD2020” gene set).

We employed two strategies to evaluate our model performance: 1) we performed gene set enrichment analysis to assess whether the top candidate genes are significantly enriched in the ASD2020 gene set; 2) we calculated area under the curve (AUC) of receiver-operating characteristic (ROC) curves (ROC-AUC) and precision-recall curves (PR-AUC). The first strategy evaluates a binary classification of risk versus non-risk genes and ignores the relative ranking of genes; the second strategy takes the ranking of genes into account and should render a more robust and comprehensive evaluation.

The ROC and PR curves in Fig. 1 show that our approach, BNScore achieves the best prediction accuracy in the ASD2020 gene set. In particular, BNScore achieves an ROC-AUC value of 0.91, higher than the other methods (with ROC-AUC values of $0.78-0.88$). The improvement over the second best method, 2020Lin, is statistically significant (one-sided Delong’s test *p* value = 0.026). Similarly, BNScore achieves a PR-AUC value of 0.68, a sizable improvement over the other methods (with PR-AUC values of $0.49{-}0.59$).

**Fig. 6:**
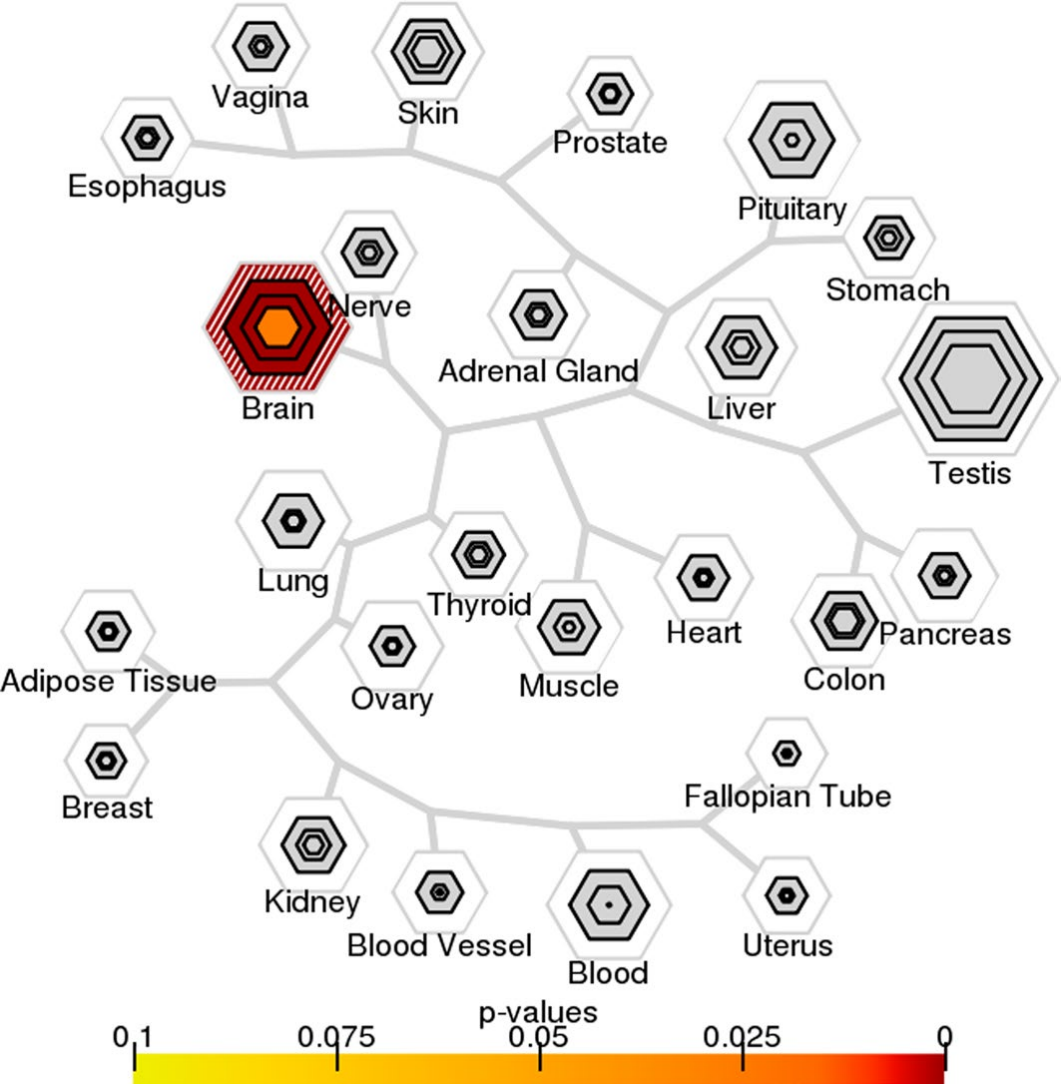
Tissue expression specificity of top candidate ASD genes. The results are reported in bulleye plots with the size of the bullseye scaled to the number of enriched genes and color coded by Fisher’s exact test *p*-values. Brain tissue is the only category of tissues significantly enriched for the top candidate genes (BH corrected *p* $\hbox {value}=1.42\times 10^{-10}$)

**Fig. 7:**
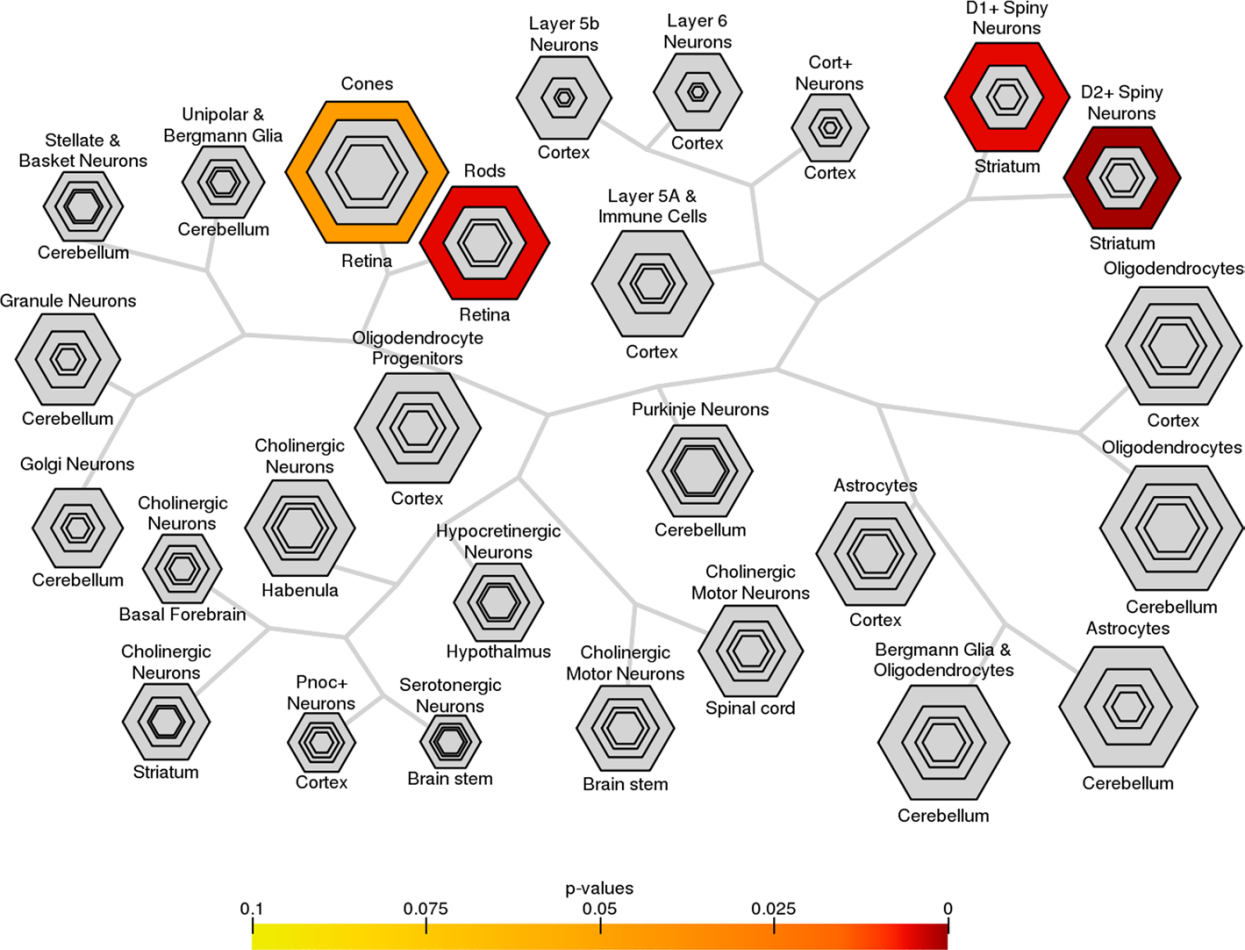
Cell type expression specificity of top candidate ASD genes. The results are reported in bulleye plots with the size of the bullseye scaled to the number of enriched genes and color coded by Fisher’s exact test *p* values. Striatal medium spiny neurons (BH corrected *p* $\hbox {value}=2.25\times 10^{-4}$) and retina (BH corrected *p* $\hbox {value}=0.05\times 10^{-1}$) are significantly enriched for top candidate genes

**Fig. 8:**
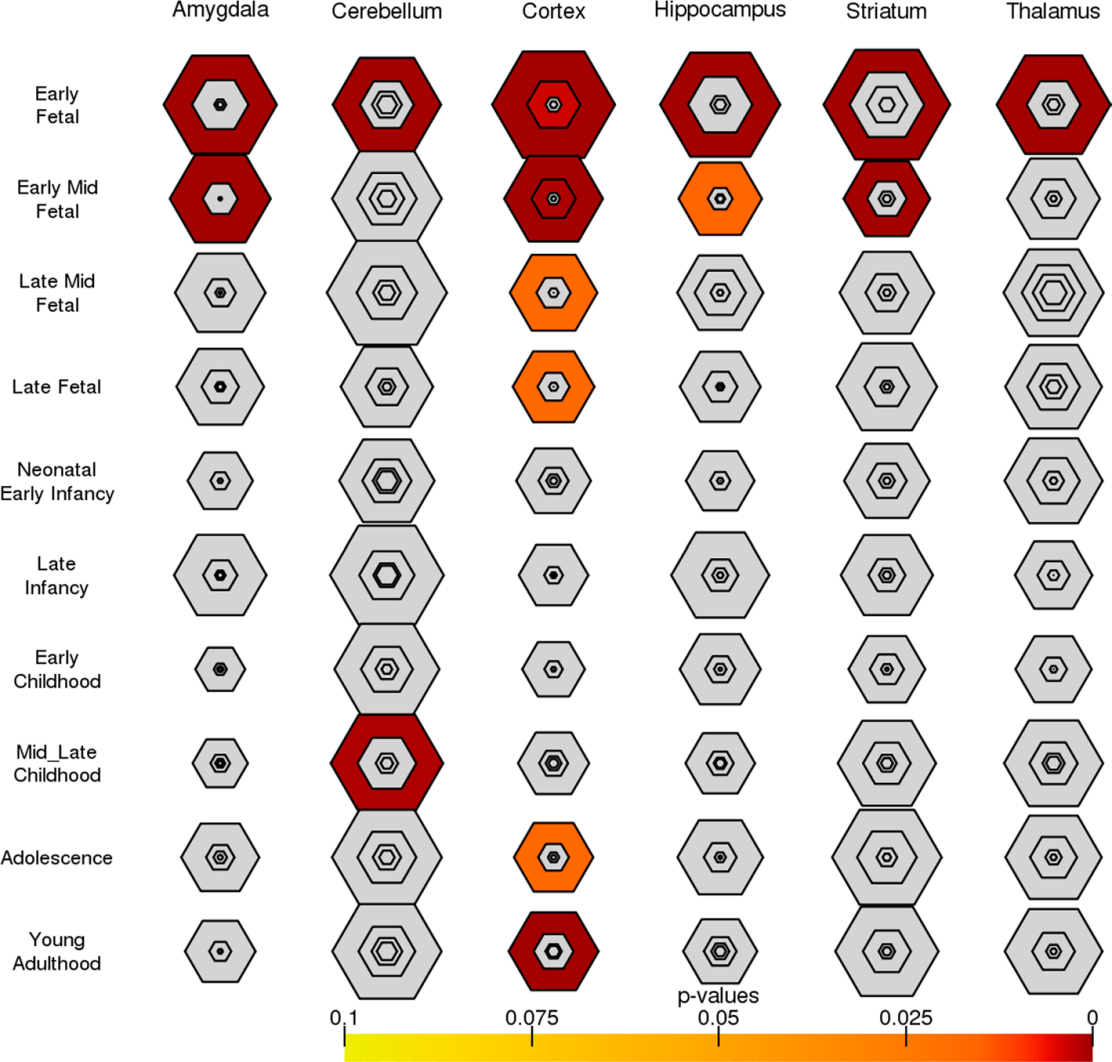
Brain region and developmental stage expression specificity of top candidate ASD genes. The results are reported in bulleye plots with the size of the bullseye scaled to the number of enriched genes and color coded by Fisher’s exact test *p*-values. Strong enrichment signals for top candidate genes are observed across all brain regions in early and mid-fetal stage, and in cerebellum and cortex during the later development stages

We tested the enrichment of the top candidate genes in the ASD2020 gene set, using the rest genes in the genome as background. Figure 2 shows that the top candidate genes predicted by BNScore are substantially more enriched in the ASD2020 gene set than the top candidated genes predicted by the other three methods. For example, the enrichment odds ratio (OR) is 24.7 (*p* $\hbox {value}=4.77\times 10^{-8}$) for the top 100 genes predicted by BNScore, compared to 18.4 (*p* $\hbox {value}=1.02\times 10^{-4}$), 14.7 (*p* $\hbox {value}=2.41\times 10^{-4}$), 14.1 (*p* $\hbox {value}=2.65\times 10^{-4}$), and 18.23 (*p* $\hbox {value}=8.60\times 10^{-4}$) for 2018Dula, 2020Lin, 2016Krishnan, and 2020Zhang respectively. The enrichment OR is 19.8 for the top 1000 genes predicted by BNScore, while the ORs are less than 15 for the other three methods. We note that genes with ranking greater than 1000 show much lower enrichment for ASD2020 genes. This is the case for all methods, indicating that most ASD risk genes are likely concentrated in the top 1000 prioritized genes, consistent with the estimate of 1000 ASD risk genes by He et al. [4]. Therefore, we focused on the top 1000 genes as the primary candidate gene sets for the rest of the study.

### Benchmarking using SFARI gene lists

We generated benchmark gene sets from the SFARI database [30], which contains genes linked to ASD from a variety of evidence sources and curated into several categories by experts. For each method, we excluded seed genes overlapped with the benchmark SFARI gene sets before evaluation. We formed three tiers of gold-standard gene sets according to the SFARI classification criteria: genes classified as “high confidence” by SFARI were designated as tier 1 evidence (T1); genes classified as “strong candidate” were designated as tier 2 evidence (T2); and genes of “suggestive evidence” were designated as tier 3 evidence (T3). We employed similar strategies as in Sect.  to compare the performance of our proposed method with existing ones.

The ROC and PR curves in Fig. 3 show that our proposed BNScore achieved the best accuracy in T1 SFARI gene set. We also tested the top 1000 candidate genes predicted by all methods for enrichment in the SFARI gene lists. Figure 4 shows that the top candidate genes predicted by BNScore are more enriched for T1 genes than the top candidate genes predicted by the other methods. The enrichment OR is 26.9 (*p*-value $=1.37\times 10^{-65}$) for BNScore, compared to 22.9 (*p* $\hbox {value}=1.50\times 10^{-45}$, 13.9 (*p* $\hbox {value}=1.28\times 10^{-26}$), 9.6 (*p* $\hbox {value}=3.82\times 10^{-14}$), and 11.7 (*p* $\hbox {value}=1.01\times 10^{-26}$) for 2020Lin, 2018Duda, 2020Zhang, and 2016Krishnan, respectively.

The AUCs for the ROC and PR curves for all methods are lower in the T2 and T3 gene sets compared to their counterparts in the T1 set (Additional file 1: Fig. S1), and the enrichment ORs are much smaller in the T2 and T3 sets compared to those in the T1 set (Additional file 1: Fig. S2). Furthermore, we did not observe significant enrichment in any SFARI gene set for genes ranked greater than 2000 by any method. This suggests that the T2 and T3 gene sets may contain a larger proportion of non-ASD genes than the T1 set. Consequently, the relative performance of the methods in T2 and T3 sets may not be as trustworthy as that in the T1 set.

### Benchmarking using ASD GWAS

We used partitioned linkage disequilibrium score regression (LDSC) [31, 32] to assess common SNP heritability enrichment near or within the top candidate genes using summary statistics from the most recent ASD GWAS [33]. Partitioned LDSC is a method to estimate the proportion of genome-wide SNP-heritability attributable to a SNP set, referred to as an “annotation” [32], while taking into account all other annotations. We annotated SNPs that are within 10 kb to the transcription start sites of the top candidate genes [34], and then used partitioned LDSC to evaluate whether these SNPs have enriched ASD heritability. Template files and code to construct annotations were adapted from the LDSC Github repository [35]. We restricted the analysis to Hapmap3 SNPs according to the recommendations from the LDSC authors. Figure 5 shows that the top candidate genes predicted by BNScore, 2016Krishnan, and 2020Lin are significantly enriched for ASD heritability. For the top 1000 genes, the enrichment OR is 7.3 (*p* $\hbox {value}=0.0068$) for BNScore, higher than that of 2020Lin (OR = 6.1, *p* $\hbox {value}=0.036$) and 2016Krishnan (OR = 6.9, *p* $\hbox {value}=0.025$). We did not include the top candidate genes predicted by 2018Duda or 2020Zhang in Fig. 5 because they were not significantly enriched for ASD heritability (e.g., *p* $\hbox {value}=0.53$, *p* $\hbox {value}=0.58$ for 2018Duda, 2020Zhang top 1000 genes, respectively).

### Examining expression specificity of top candidate genes

We explored the tissue, cell type, and brain developmental stage specificity of the prioritized ASD risk genes to further dissect the ASD etiology. To this end, we calculated the gene expression specificity with respect to tissues, cell types, and brain developmental stages based on transcriptome data using the specificity index (*pSI*) developed by Dougherty et al. [26]. We tested our top 1000 candidate ASD risk genes for enrichment in each tissue’s, cell type’s, or brain developmental stage’s specific gene list using Fisher’s exact test. We used the Benjamini-Hochberg (BH) procedure to control the false discovery rate [36].

To evaluate tissue specificity, we used the Genotype-Tissue Expression (GTEx) dataset [37]. For tissues with multiple replicates, we averaged reads per kilobase of transcript per million mapped reads values before use. For each tissue, we defined a list of specifically expressed genes by selecting genes with $pSI<0.05$ (the smaller the more specific). As shown in Fig. 6, we found the brain tissues to be the only category of tissues significantly enriched for the top candidate genes (BH corrected $p-\hbox {value}=1.42\times 10^{-10}$).

To study cell type specificity, we used the mice datasets published by Xu et al. [38]. For each cell type, we defined a list of specifically expressed genes by selecting genes with $pSI<0.05$. Figure 7 shows the over-representation of candidate genes in striatal medium spiny neurons and retina specific genes. Defects in the striatum have previously been found to specifically contribute to the motor, social, and communication impairments seen in ASD patients [39, 40], while retina has been used as an accessible window to understand brain wiring and functions as it uses and produces most neurotransmitters found in the brain [41].

To study spatiotemporal expression patterns in human brain, we used the Brainspan dataset [42], which was condensed into six major regional divisions across ten developmental stages. For each brain region and developmental stage, we defined a list of specifically expressed genes by selecting genes with $pSI<0.05$. Figure 8 shows strong enrichment signals for our candidate ASD risk genes in early and mid-fetal stage genes across all brain regions. We observed only a few enrichment signals in the later development stages, e.g., in cerebellum during mid-late childhood and in cortex during young adulthood. These findings are consistent with the reported heterogeneity of ASD, as abruptions from many brain regions across many developmental stages may all contribute to the onset of ASD [8, 43].

## Discussion

We present BNScore, a Bayesian model selection based framework to facilitate genome-wide ASD gene discovery. The Bayesian modeling frameword has advantages in interpretability of final results compared to hypothesis-testing approaches and machine learning algorithms. Our approach is flexible in integrating multiple types of biological evidence. Currently, it integrates sequencing study results, diverse functional annotations, and network information to obtain genome-wide prediction of ASD risk genes. It is straightforward to incorporate new lines of evidence as they become available in the future.

Our prediction is validated by three benchmark datasets not used in the training process: (1) a recently published exome sequencing study [28], (2) genes from the SFARI database, and (3) a recently published ASD GWAS study [33]. Our approach outperforms the existing methods in most situations, pinpointing 1000 top candidate genes with high confidence. We observe that our top candidate genes are specifically expressed in brain tissues, in striatal medium spiny neurons and retina, and in early developmental stages across brain regions, offering hypotheses for further validation of the implicated tissue, cell types, and developmental stages.

The performance gain for BNScore relative to the other approaches may be attributed to the integration of a variety types of biological evidence, including sequencing study results, diverse functional annotations, and network information to prioritize ASD risk genes. The data integration allows us to take advantage of the complementary information contained in them to improve the prediction of ASD risk genes. Also, genes with multiple sources of evidence pointing to them might be more likely to be true risk genes [44]. In fact, the enrichment ORs of the top 1000 genes ranked using network distance only, Bayes factor based on binary annotations only, Bayes factor based on continuous annotations only are 3.34 (*p* $\hbox {value}=1.9\times 10^{-2}$), 13.09 (*p* $\hbox {value}=3.26\times 10^{-17}$), 17.28 (*p* $\hbox {value}=6.16\times 10^{-22}$), respectively, which are all smaller than the OR of the final BNScore model (OR = 19.83, *p* $\hbox {value}=1.82\times 10^{-24}$) for ASD2020 genes.

There are other types of evidence that may be useful for ASD risk gene prioritization. For example, epigenomics data that have been implicated in ASD risk genes may provide additional important evidence [45] , and phenome networks may provide more evidence for gene-gene connections [46]. Our current analysis is limited to protein-coding genes. Recent studies have also shown that long non-coding RNAs (lncRNA), which are important regulators of gene expression, could also be prioritized for ASD risk through transferring knowledge from protein-coding genes [47]. Given the flexibility of our framework, these aspects could be readily explored in future studies and might contribute to further improvement in prediction accuracy.

We assumed parametric models in the Bayes factor calculations because of their ease of implementation and computational efficiency. These models work well in our empirical datasets. In the future, one may encounter other types of annotations that cannot be appropriately represented by these simple models. In this situation, one may consider more sophisticated semiparametric or nonparametric models. In general, the advantage of Bayesian modeling lies in its ability to incorporate prior probabilities for the data generating mechanism, isolate the effect of each feature, and identify parameters that are interpretable and of special interest (e.g. $\theta _{ij}$ for binary annotation in our approach). As a contrast, machine learning models usually does not attempt to isolate the effect of any single variable and usually does not model the data generating process but instead attempt to learn from the dataset through intractable processes. With that being said, we acknowledge that there are scenarios where machine learning methods can be preferable, e.g., in the presence of complicated nonlinear interactions, when the dataset is huge, or when overall prediction is the only goal and there is no need to succinctly describe the impact of any one variable.

The performance of our proposed BNScore, as well as any other method for ASD gene prioritization, ultimately depends on the seed ASD genes, which may not be representative of the full spectrum of ASD risk genes. In other words, our approach is more powerful to identify new ASD candidate genes similar to “known” disease genes. Therefore, we interpret our results with caution: we can implicate candidate genes but we are not confident to exclude genes that are not similar to seed genes, since such genes may lead to diseases through entirely unexpected mechanisms yet represented in the seed genes.

Like other gene prioritization approaches, our approach is based on the available incomplete annotation data sources, which themselves incorporate false positive/negative annotations and bias studies of human genome. The prioritized genes offer relevant hypotheses to researchers to further investigate.

## Conclusion

Overall, owing to the benefits from integrating sequencing findings, functional annotation profiles, and gene-gene functional network, our approach provides competitive performance compared to current state-of-the-art methods when validated in benchmark datasets. The Bayesian setup provides easily interpretable results. With the expansion of both genomic data and epigenomic data in the future, the identification of risk genes could be further improved by expanding our framework to include more annotations. Although designed for ASD, we note that this approach can be extended to other complex traits. It is our hope that this framework can offer prioritized risk genes to researchers to facilitate the identification of disease risk genes.

## Supplementary Information

**Additional file 1:** Supplementary Notes, Tables and Figures.

## Data Availability

The datasets generated and code are available in the GitHub repository https://github.com/yingji15/ASD_public.

## Abbreviations

ASD: Autism spectrum disorder
ROC-AUC: Area under the receiver-operating characteristic (ROC) curve
PR-AUC: Area under the precision-recall curve
SFARI: Simons foundation autism research initiative
OR: Odds ratio
SI: Specificity index
LDSC: Linkage disequilibrium score regression
