# Supplementary material for: A Bayesian framework to integrate multi-level genome-scale data for Autism risk gene prioritization
Source: BMC Bioinformatics. 2022 Apr 22;23:146. doi: 10.1186/s12859-022-04616-y (PMC9034518; doi:10.1186/s12859-022-04616-y)
Supplement: Supplementary file 1 — Additional file 1: Supplementary Notes, Tables and Figures. [file 12859_2022_4616_MOESM1_ESM.pdf]

A Bayesian framework to integrate multi-level genome-scale data for Autism risk gene prioritization: supplemental document

1. SUPPLEMENTARY NOTES

A. Prior odds that a gene being an ASD risk gene: network construction

We follow previous work [1, 2] to construct a gene-gene network to represent each gene's prior odds of being an ASD gene. The following are the three major steps in this process.

A.1. Construct an adjacency matrix from Gene Ontology (GO) annotations

We created a weighted network from publicly available GO annotations. Each GO annotation term includes a gene product and a molecular function, biological process, or cellular component. First, we assign a weight for each GO annotation term T (e.g., a molecular function) based on how specific the term is: measured by the number of genes associated with the term (N_T) divided by total number of genes (N_{total}) with annotations.

$$S_T = \frac{N_T}{N_{total}} \tag{S1}$$

Then, for distance between gene i (g_i) and gene j (g_j), we assign a score proportional to the log of the ratio of the likelihood that the two genes participate in the same GO annotation to the likelihood that they do not by summing all the terms shared by these two genes [1].

$$W_{g_i,g_j} = \sum_{g_i,g_j \in T} -2log(S_T) \tag{S2}$$

Then we column normalized matrix W to make them unit-length, and used the resulting matrix W as adjacency matrix.

A.2. Construct a transition matrix from adjacency matrix

From the symmetric adjacency matrix W, we derive a transition matrix P. The rows of the transition matrix are probability vectors, in other words, the rows of matrix P are numerical vectors whose entries are real numbers between 0 and 1 whose sum is 1. We use P_{g_i,g_j} to reflect the probability of stepping to node j from node i informed by the adjacency matrix W.

$$P_{g_i,g_j} = \frac{W_{g_i,g_j}}{\sum_i W_{g_i,g_j}} \tag{S3}$$

A.3. Apply random walk with restart algorithm to compute distance

Here we use Random walk with restart (RWR) to measure how closely related are two genes (i.e. nodes in a weighted graph) in the transition matrix P ([2, 3]). For each step starting from any node i of the network, the walker has two options: either move to a neighbor with a probability $1 - r$ or stay at node i with a probability r. The parameter r is called the restart probability. In the current study, we fix $r = 0.3$. The probability of the walker to walk to each neighbor is proportional to the weight of the edge connecting

them. Let q_t denote a vector with the reaching probability to all nodes at step t starting from node i.

$$q_{t+1} = (1-r)Pq_t + rS_i \tag{S4}$$

S_i is an indicator vector with the ith element as 1 and 0 for others, which means the starting node is node i. q_t can be updated step by step until $|q_{t+1} - q_t| < T$, where T is a predefined threshold. We set T to $1e^{-6}$ in this study.

For each gene i, let q_t be the vector with the reaching probabilities to all the genes (after stabilization), let $P(N_p)$ be the average reaching probabilities to all positive training genes, which is proportional to the prior odds of gene i being a risk gene.

B. Bayes factor derivations

B.1. Binary annotations

We assume that a binary annotation D_l follows the Bernoulli distribution $Bernoulli(\theta_{lj})$, where θ_{lj} represents the fraction of disease-associated genes with this annotation under model M_j $(j = 0, 1)$.

To estimate the parameter θ_{ij}, suppose we have a prior distribution of the parameter (determined by hyperparameters of beta distribution), and we observe the training set genes along with their annotations information and labels (disease-associated/non-disease-associated), we use empirical Bayes approach to estimate the distribution of these parameters and hyperparameters.

- Prior distribution of θ_{ij}

  To obtain conjugate prior for θ, we use Beta distribution here.

  Under M_j: $\theta_{lj}|\alpha_{lj}, \beta_{lj} \sim Beta(\alpha_{lj}, \beta_{lj})$

  $$f(\theta_{lj}|\alpha_{lj}, \beta_{lj}) = \frac{1}{B(\alpha_{lj}, \beta_{lj})} \theta_{lj}^{\alpha_{lj}-1} (1-\theta_{lj})^{\beta_{lj}-1}$$

  with $0 < \theta_{lj} < 1$

- Posterior distribution of θ_{ij}

  From the training data, we can update the distribution of θ_{ij}

  Suppose we have n seed genes, k of which possess this binary annotation, i.e., $\sum_{g=1}^{n} D_{lg} = k$.

  For a single observation, the marginal likelihood is

  $$\begin{aligned} p(D_{l1} = k|\alpha_{l1}, \beta_{l1}) &= \int p(D_{l1} = k|\theta_{l1}) p(\theta_{l1}|\alpha_{l1}, \beta_{l1}) d\theta_{l1} \\ &= \frac{B(\alpha_{l1} + k, n - k + \beta_{l1})}{B(\alpha_{l1}, \beta_{l1})} \end{aligned}$$

  The moments of Beta-Bernoulli distributions are:

  $$E(D_l) = \frac{\alpha_{l1}}{\alpha_{l1} + \beta_{l1}}$$

  $$Var(D_l) = \frac{\alpha_{l1}\beta_{l1}}{(\alpha_{l1} + \beta_{l1})^2}$$

  Let the first moment of D be $A_1 = \frac{1}{n}\sum_{g=1}^{n} D_l^k$ and we equate $E(D_l)$ to A_1. As we have one equation and two parameters to estimate, we assume $\tilde{\beta_{i1}} = 1$ so

that we can get estimate $\tilde{\alpha_{i1}}$ by solving $E(D_l) = A_1$, the resulting estimate for hyperparameters:

$$\tilde{\beta_{l1}} = 1$$

$$\tilde{\alpha_{l1}} = \frac{A_1}{1 - A_1}$$

With these in hand, the estimated mean of θ_{l1}:

$$E(\tilde{\theta_{l1}}) = \frac{\tilde{\alpha_{l1}}}{\tilde{\alpha_{l1}} + \tilde{\beta_{l1}}} \tag{S5}$$

Similarly, under M_0, we can derive the posterior estimate of α_{i0} and β_{i0} from background genes.

B.2. Continuous annotations

We assume that a continuous annotation D_l follows a normal distribution $N(\mu_{lj}, \theta_{lj})$, where μ_{lj} and θ_{lj} are the mean and variance, respectively, under model M_j $(j = 0, 1)$.

- Prior distribution of parameters

  Let $\eta_{lj} = (\mu_{0lj}, \kappa_{lj}, v_{lj}, \sigma^2_{lj})^{\mathrm{T}}$ denote the hyperparameters.

  $\mu_{lj}|\theta_{lj} \sim N(\mu_{0lj}, \frac{\theta_{lj}}{\kappa_{lj}}); \theta_{lj} \sim IG(v_{lj}/2, v_{lj}\sigma^2_{lj}/2)$.

- Posterior distribution of parameters

  We compute the marginal distribution of D_l following [4] under model M_j. It can be shown that D_l follows a non-standardized t-distribution following the steps below:

$$p(D_l|\eta_{lj}) = \frac{f(D_l|\mu_{lj}, \theta_{lj})\pi(\mu_{lj}, \theta_{lj}|\eta_{lj})}{\pi(\mu_{lj}, \theta_{lj}|D_l, \eta_{lj})}$$

  To lighten notations, η_{lj} and subscripts lj will be dropped in the densities below (i.e, μ_0 for μ_{0lj},κ for κ_{lj}):

$$\pi(\mu, \theta|D, \eta) \propto f(D|\mu, \theta)\pi(\mu, \theta)$$

$$\pi(\mu, \theta) = \pi(\mu|\theta)\pi(\theta)$$

$$\pi(\mu, \theta|D) \propto f(D|\mu, \theta)\pi(\mu|\theta)\pi(\theta)$$

  We have:

$$f(D|\mu, \theta) = \frac{1}{\sqrt{2\pi\theta}}exp(-\frac{(D-\mu)^2}{2\theta}) \propto (\frac{1}{\theta})^{1/2}exp(-\frac{1}{2\theta}(D-\mu)^2$$

  Thus,

$$\pi(\mu, \theta|D) \propto \theta^{\frac{-1}{2}}\theta^{-\frac{v}{2}+1}exp\{-\frac{1}{2\theta}[(x-\mu)^2 + \kappa(\mu-\mu_0)^2 + v\sigma^2]\}$$

  Denote $\mu_n = \frac{x+\mu_0}{1+\kappa}$, $\kappa_n = \kappa + 1$, $v_n = v + 1$, $v_n\sigma^2_n = v\sigma^2 + \frac{\kappa}{1+\kappa}(x-\mu_0)^2$, we have

$$(x-\mu)^2 + \kappa(\mu-\mu_0)^2 + v\sigma^2 = \kappa_n(\mu-\mu_n)^2 + v_n\sigma^2_n$$

The above have shown that $\pi(\mu, \theta|x)$ is a normal-inverse-gamma distribution: $\mu|\theta, D \sim N(\mu_n, \theta/2)$, $\theta|D \sim IG(\alpha^*, \beta^*)$, With $\alpha^* = \frac{v_n}{2}, \beta^* = \frac{v_n \sigma_n^2}{2}$. Then we can show

$$\begin{aligned} f(D|\mu,\theta)\pi(\mu,\theta) &= f(D|\mu,\theta)\pi(\mu|\theta)\pi(\theta) \\ &= (2\pi)^{-1}\sqrt{\kappa}\frac{(\frac{v\sigma^2}{2})^{v/2}}{\Gamma(\frac{v}{2})} \\ &\times \theta^{-\frac{1}{2}}\theta^{-(\frac{v_n}{2}+1)} exp\{-\frac{1}{2\theta}[\kappa_n(\mu-\mu_n)^2 + v_n\sigma_n^2]\} \end{aligned}$$

Denote

$$C_1 = (2\pi)^{-1}\sqrt{\kappa}\frac{(\frac{v\sigma^2}{2})^{v/2}}{\Gamma(\frac{v}{2})}$$

The joint posterior distribution:

$$\begin{aligned} \pi(\mu,\sigma|D) &= \pi(\mu|\theta,D)\pi(\theta|D) \\ &= \frac{\sqrt{\kappa_n}}{\sqrt{2\pi}}\frac{(\frac{v\tilde{\sigma}^2}{2})^{v/2}}{\Gamma(\frac{v}{2})} \\ &\times \theta^{-\frac{1}{2}}\theta^{-(\frac{v_n}{2}+1)} exp\{-\frac{1}{2\theta}[\kappa_n(\mu-\mu_n)^2 + v_n\sigma_n^2]\} \end{aligned}$$

Denote

$$C_2 = \frac{\sqrt{\kappa_n}}{\sqrt{2\pi}}\frac{(\frac{v_n\sigma_n^2}{2})^{v_n/2}}{\Gamma(\frac{v_n}{2})}$$

Consequently,

$$\begin{aligned} p(D|\eta) &= \frac{f(D|\mu,\theta)\pi(\mu,\theta|\eta)}{\pi(\mu,\theta|D,\eta)} \\ &= \frac{C_1\theta^{-1/2}\theta^{-\frac{v_n}{2}+1} exp\{-\frac{1}{2\theta}[\kappa_n(\mu-\mu_n)^2 + v_n\sigma_n^2]\}}{C_2\theta^{-1/2}\theta^{-\frac{v_n}{2}+1} exp\{-\frac{1}{2\theta}[\kappa_n(\mu-\mu_n)^2 + v_n\sigma_n^2]\}} = \frac{C_1}{C_2} \end{aligned}$$

Given a gene with value D for the continuous annotation, we have $n = 1$, and the marginal probability becomes:

$$p(D|\eta) = \frac{C_1}{C_2} \qquad \propto [\frac{v\sigma^2 + \frac{\kappa}{1+\kappa}(D-\mu_0)^2}{2}]^{-\frac{v+1}{2}} \sim t_v(\mu_0, \frac{(1+\kappa)\sigma^2}{\kappa}),$$

which is a non-standardized t-distribution.

For $D \sim t_v(\mu, \sigma^2)$, it was shown that the first six moments of D are [5]:

$$ED = \mu$$

if $v > 2$:

$$ED^2 = \mu^2 + \frac{v\sigma^2}{v-2}$$

$$ED^3 = \mu^3 + 3\mu\frac{v\sigma^2}{v-2}$$

if $v > 4$:

$$ED^4 = \mu^4 + 6\mu^2 \frac{v\sigma^2}{v-2} + 3\frac{v^2\sigma^4}{(v-2)(v-4)}$$

$$ED^5 = \mu^5 + 10\mu^3 \frac{v\sigma^2}{v-2} + 15\mu\frac{v^2\sigma^4}{(v-2)(v-4)}$$

if $v > 6$:

$$ED^6 = \mu^6 + 15\mu^4 \frac{v\sigma^2}{v-2} + \\ 45\mu^2 \frac{v^2\sigma^4}{(v-2)(v-4)} + 15\frac{v^3\sigma^6}{(v-2)(v-4)(v-6)}$$

Here we can obtain estimators for μ if we set $\tilde{\kappa} = 1$

We use the posterior distribution of D and method of moments to estimate prior parameters under M_1 using seed genes and parameters under M_0 using background genes. Let $A_k = \frac{1}{n}\sum_{g=1}^{n} D_g^k$ (kth moment of D), we have:

$$\tilde{\mu_0} = A_1$$

$$\tilde{\kappa} = 1$$

$$\tilde{v} = \frac{-\frac{14}{3}A_1^4 + 4A_1^2A_2 + 2A_2^2 - \frac{4}{3}A_4}{-\frac{2}{3}A_1^4 + A_2^2 - \frac{1}{3}A_4}$$

$$\tilde{\sigma^2} = \frac{-\frac{1}{6}(A_2 - A_1^2)(5A_1^4 - 6A_1^2A_2 + A_4)}{-\frac{7}{3}A_1^4 + 2A_1^2A_2 + A_2^2 - \frac{2}{3}A_4}$$

2. SUPPLEMENTARY TABLE

Table S1. Annotations included in the framework

Gene set	Short description
EG ([6])	essential genes (human orthologs of genes with an essential role)
HIS ([7])	Haploinsufficiency Score (probability of being haploinsufficient)
GHIS ([8])	Genome-Wide Haploinsufficiency Score
FMRP_target ([9])	Fragile X mental retardation (FMRP) protein targets
pLI ([10])	probability of being loss-of-function intolerant
RVIS_maf_0.05. ([11])	Genic Intolerance to Functional Variation (variants of MAF $>$ 0.05)
OMIM ([11, 12])	OMIM disease genes
ncRVIS ([13])	noncoding region genic Intolerance to Functional Variation
embryonic ([14, 15])	genes expressed preferentially in embryos
chromatin_modi ([16])	genes encoding chromatin modifiers
rbfox ([17])	Rbfox Splicing-Regulatory Network target genes
ca ([18])	Calcium channel and signaling genes
miR-137 ([19])	miR-137 target genes
presynapse ([20])	presynaptic genes
CHD8 ([21])	chromatin remodeler CHD8 regulated genes
PSD-95 ([22])	post synaptic genes
his_mod_enz ([16])	histone modifying enzymes

3. SUPPLEMENTARY FIGURES

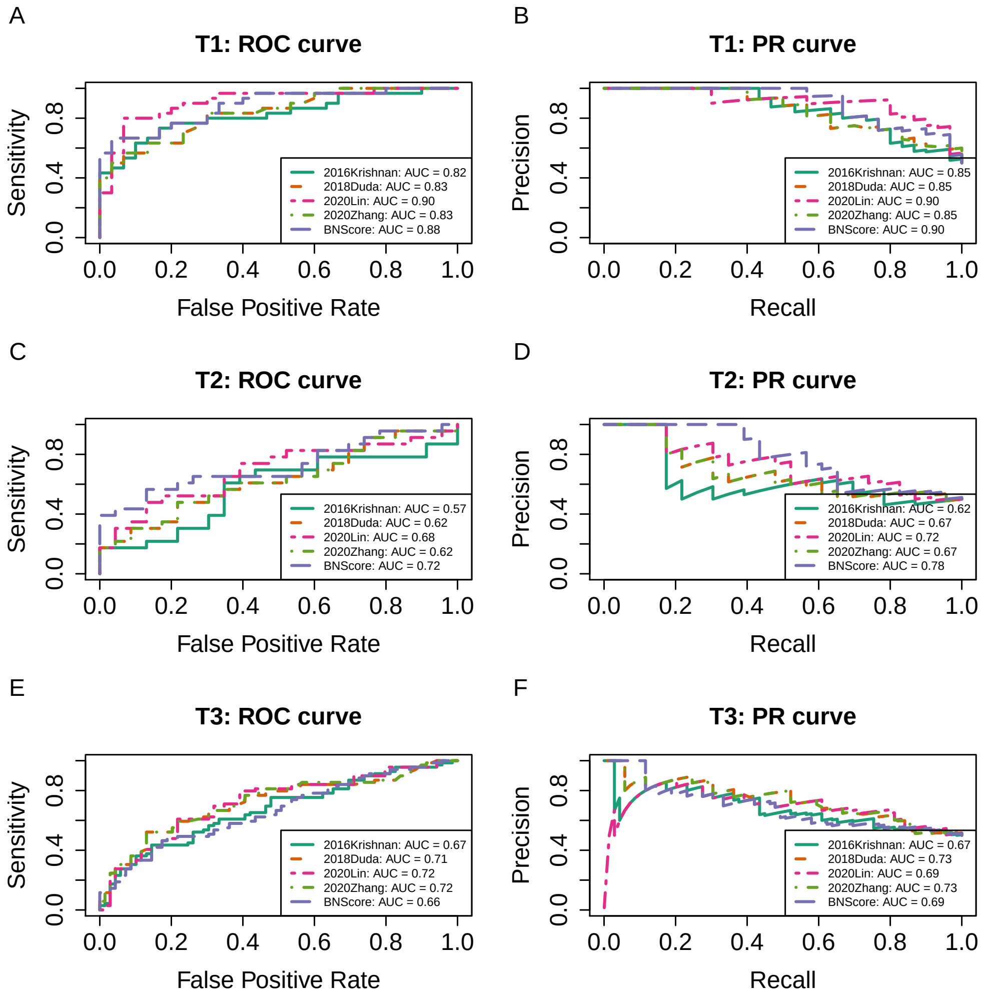

Fig. S1. ROC and PR curves of the five methods in the SFARI gene sets.

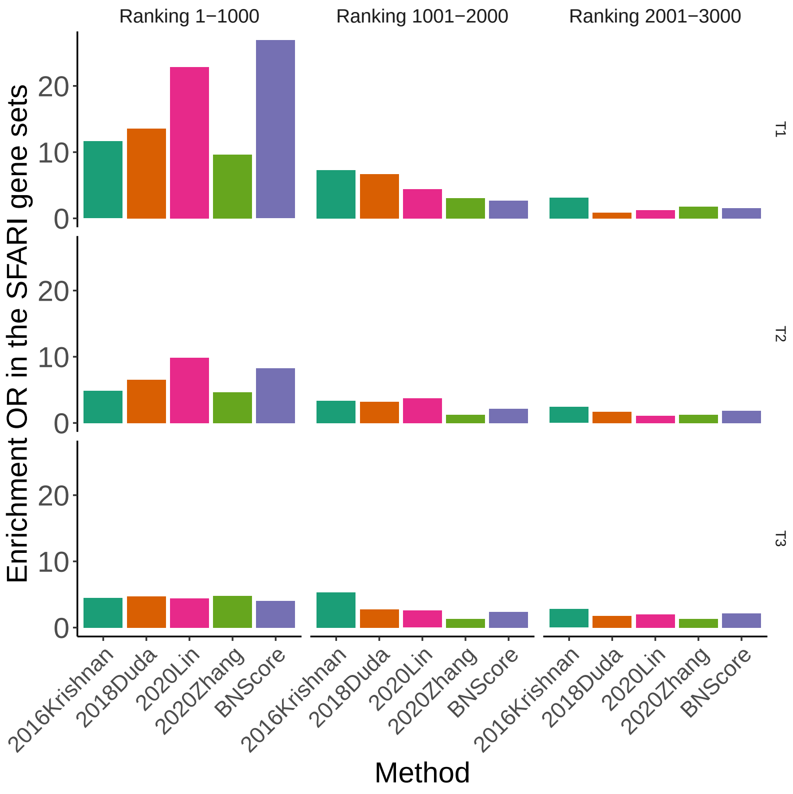

Fig. S2. Enrichment of top candidate genes in the SFARI gene sets.

REFERENCES

1. S. R. Gilman, I. Iossifov, D. Levy, M. Ronemus, M. Wigler, and D. Vitkup, "Rare de novo variants associated with autism implicate a large functional network of genes involved in formation and function of synapses," Neuron **70**, 898–907 (2011).
2. Q. Wang, R. Chen, F. Cheng, Q. Wei, Y. Ji, H. Yang, X. Zhong, R. Tao, Z. Wen, J. S. Sutcliffe *et al.*, "A bayesian framework that integrates multi-omics data and gene networks predicts risk genes from schizophrenia gwas data," Nat. neuroscience **22**, 691–699 (2019).
3. H. Tong, C. Faloutsos, and J.-Y. Pan, "Random walk with restart: fast solutions and applications," Knowl. Inf. Syst. **14**, 327–346 (2008).
4. Y.-Y. Zhang, T.-Z. Rong, and M.-M. Li, "The empirical bayes estimators of the mean and variance parameters of the normal distribution with a conjugate normal-inverse-gamma prior by the moment method and the mle method," Commun. Stat. Methods **48**, 2286–2304 (2019).
5. Y. Zhang, S. Qiao, L. Sun, Q. W. Shi, W. Huang, L. Li, and Z. Yang, "Photoinduced active terahertz metamaterials with nanostructured vanadium dioxide film deposited by sol-gel method," Opt. Express **22**, 11070–11078 (2014).
6. X. Ji, R. L. Kember, C. D. Brown, and M. Bućan, "Increased burden of deleterious variants in essential genes in autism spectrum disorder," Proc. Natl. Acad. Sci. **113**, 15054–15059 (2016).
7. N. Huang, I. Lee, E. M. Marcotte, and M. E. Hurles, "Characterising and predicting haploinsufficiency in the human genome," PLoS Genet. **6**, e1001154 (2010).
8. J. Steinberg, F. Honti, S. Meader, and C. Webber, "Haploinsufficiency predictions without study bias," Nucleic Acids Res. **43**, e101–e101 (2015).
9. J. C. Darnell, S. J. Van Driesche, C. Zhang, K. Y. S. Hung, A. Mele, C. E. Fraser, E. F. Stone, C. Chen, J. J. Fak, S. W. Chi *et al.*, "Fmrp stalls ribosomal translocation on mrnas linked to synaptic function and autism," Cell **146**, 247–261 (2011).
10. M. Lek, K. J. Karczewski, E. V. Minikel, K. E. Samocha, E. Banks, T. Fennell, A. H. O'Donnell-Luria, J. S. Ware, A. J. Hill, B. B. Cummings *et al.*, "Analysis of protein-coding genetic variation in 60,706 humans," Nature **536**, 285–291 (2016).
11. S. Petrovski, Q. Wang, E. L. Heinzen, A. S. Allen, and D. B. Goldstein, "Genic intolerance to functional variation and the interpretation of personal genomes," PLoS Genet. **9**, e1003709 (2013).
12. A. Hamosh, A. F. Scott, J. S. Amberger, C. A. Bocchini, and V. A. McKusick, "Online mendelian inheritance in man (omim), a knowledgebase of human genes and genetic disorders," Nucleic Acids Res. **33**, D514–D517 (2005).
13. S. Petrovski, A. B. Gussow, Q. Wang, M. Halvorsen, Y. Han, W. H. Weir, A. S. Allen, and D. B. Goldstein, "The intolerance of regulatory sequence to genetic variation predicts gene dosage sensitivity," PLoS Genet. **11**, e1005492 (2015).
14. I. Voineagu, X. Wang, P. Johnston, J. K. Lowe, Y. Tian, S. Horvath, J. Mill, R. M. Cantor, B. J. Blencowe, and D. H. Geschwind, "Transcriptomic analysis of autistic brain reveals convergent molecular pathology," Nature **474**, 380–384 (2011).
15. H. J. Kang, Y. I. Kawasawa, F. Cheng, Y. Zhu, X. Xu, M. Li, A. M. Sousa, M. Pletikos, K. A. Meyer, G. Sedmak *et al.*, "Spatio-temporal transcriptome of the human brain," Nature **478**, 483–489 (2011).
16. I. Iossifov, B. J. O'roak, S. J. Sanders, M. Ronemus, N. Krumm, D. Levy, H. A. Stessman, K. T. Witherspoon, L. Vives, K. E. Patterson *et al.*, "The contribution of de novo coding mutations to autism spectrum disorder," Nature **515**, 216–221 (2014).
17. S. M. Weyn-Vanhentenryck, A. Mele, Q. Yan, S. Sun, N. Farny, Z. Zhang, C. Xue, M. Herre, P. A. Silver, M. Q. Zhang *et al.*, "Hits-clip and integrative modeling define the rbfox splicing-regulatory network linked to brain development and autism," Cell Rep. **6**, 1139–1152 (2014).
18. C.-D. G. of the Psychiatric Genomics Consortium *et al.*, "Identification of risk loci

with shared effects on five major psychiatric disorders: a genome-wide analysis," Lancet **381**, 1371–1379 (2013).

19. E. Mahmoudi and M. Cairns, "Mir-137: an important player in neural development and neoplastic transformation," Mol. psychiatry **22**, 44–55 (2017).
20. M. Pirooznia, T. Wang, D. Avramopoulos, D. Valle, G. Thomas, R. L. Huganir, F. S. Goes, J. B. Potash, and P. P. Zandi, "Synaptomedb: an ontology-based knowledgebase for synaptic genes," Bioinformatics **28**, 897–899 (2012).
21. A. L. Gompers, L. Su-Feher, J. Ellegood, N. A. Copping, M. A. Riyadh, T. W. Stradleigh, M. C. Pride, M. D. Schaffler, A. A. Wade, R. Catta-Preta *et al.*, "Germline chd8 haploinsufficiency alters brain development in mouse," Nat. Neurosci. **20**, 1062 (2017).
22. À. Bayés, L. N. Van De Lagemaat, M. O. Collins, M. D. Croning, I. R. Whittle, J. S. Choudhary, and S. G. Grant, "Characterization of the proteome, diseases and evolution of the human postsynaptic density," Nat. Neurosci. **14**, 19–21 (2011).
